# Exploring the Impact of Different Types of Do-Not-Resuscitate Consent on End-of-Life Treatments among Patients with Advanced Kidney Disease: An Observational Study

**DOI:** 10.3390/ijerph18158194

**Published:** 2021-08-02

**Authors:** Chiu-Hsien Yang, Chien-Yi Wu, Joseph T. S. Low, Yun-Shiuan Chuang, Yu-Wen Huang, Shang-Jyh Hwang, Ping-Jen Chen

**Affiliations:** 1 Department of Family Medicine, Kaohsiung Medical University Hospital, Kaohsiung Medical University, Kaohsiung 807, Taiwan; 9999yang@gmail.com (C.-H.Y.); dietcokewu0822@gmail.com (C.-Y.W.); kinkipag@gmail.com (Y.-S.C.); 2 Marie Curie Palliative Care Research Department, Division of Psychiatry, University College London, London W1T 7NF, UK; joseph.low@ucl.ac.uk; 3 Department of Nursing, Kaohsiung Medical University Hospital, Kaohsiung Medical University, Kaohsiung 807, Taiwan; carey04292000@yahoo.com.tw; 4 Division of Nephrology, Department of Internal Medicine, Kaohsiung Medical University Hospital, Kaohsiung Medical University, Kaohsiung 807, Taiwan; sjhwang@kmu.edu.tw; 5 School of Medicine, College of Medicine, Kaohsiung Medical University, Kaohsiung 807, Taiwan

**Keywords:** palliative care, end-of-life care, chronic kidney failure, resuscitation orders, patient autonomy, advance care planning

## Abstract

*Background*: Patients with advanced kidney disease have a symptomatic and psychological burden which warrant renal supportive care or palliative care. However, the impact of do-not-resuscitate consent type (signed by patients or surrogates) on end-of-life treatments in these patients remains unclear. *Objective*: We aim to identify influential factors correlated with different do-not-resuscitate consent types in patients with advanced kidney disease and the impact of do-not-resuscitate consent types on various life-prolonging treatments. *Methods:* This was a retrospective observational study. We included patients aged 20 years and over, diagnosed with advanced kidney disease and receiving palliative and hospice care consultation services between January 2014 and December 2018 in a tertiary teaching hospital in Taiwan. We reviewed medical records and used logistic regression to identify factors associated with do-not-resuscitate consent types and end-of-life treatments. *Results*: A total of 275 patients were included, in which 21% signed their do-not-resuscitate consents. A total of 233 patients were followed until death, and 32% of the decedents continued hemodialysis, 75% underwent nasogastric (NG) tube placement, and 70% took antibiotics in their final seven days of life. Do-not-resuscitate consents signed by patients were associated with reduced life-prolonging treatments including feeding tube placement and antibiotic use in the last seven days (odd ratio and 95% confidence interval were 0.16, 0.07–0.34 and 0.33, 0.16–0.69, respectively) compared to do-not-resuscitate consents signed by surrogates. *Conclusions:* Do-not-resuscitate consent signed by patients and not by surrogates may reflect better patients’ autonomy and reduced life-prolonging treatments in the final seven days of patients with advanced kidney disease.

## 1. Introduction

Global prevalence of chronic kidney disease (CKD) prevalence varies from 23–36% in people aged >64 years worldwide [1]. Taiwan reported the highest incidence and prevalence of treated end-stage renal disease (ESRD) in the world in 2016, in which over half of the patients starting hemodialysis were aged >65 years [2] and the median survival was between 1.6 to 4.6 years with different comorbidities among individuals entering hemodialysis aged >75 years [3]. In addition to the poor prognosis in older patients, the symptomatic and psychological burden of advanced kidney disease including stage 4 CKD and stage 5 CKD with or without renal replacement therapy (RRT) prompted the use of renal supportive or palliative care, which places greater emphasis on quality of life than the length of survival [4].

In Taiwan, changes in legislation and health policy have been implemented to improve the quality of end-of-life care for people with life-threatening diseases. Hospice Palliative Care Act (a Natural Death Act), which was legislated in 2000, have endorsed the right for patients to refuse cardiopulmonary resuscitation (CPR) and two types of do-not-resuscitate (DNR) documents have been formulated: (i) DNR consented by patients and the document signed by themselves and (ii) DNR decisions assented and signed by surrogates when the patient is in terminal illness and incapable of consent. In 2009, the National Health Insurance (NHI) expanded the reimbursement for palliative and hospice care to patients with nonmalignant disease, including those with acute or chronic kidney failure [5]. The Patient Right to Autonomy Act (PRAA)—legislated in 2016 and implemented in 2019—expands patients’ rights of refusing more categories of life-sustaining treatments (LST) and endorsing the right of refusal in more clinical situations other than terminally-ill status. It also provides the legal regulation for advanced care planning (ACP) and advanced directives regarding treatment preferences, such as tube feeding, artificial nutrition support, antibiotic use, and any procedures for prolonging life.

Advanced directives reflect patients’ autonomy and may be better than surrogate decision-making. The patients must be cognitively competent and motivated to express their end-of-life preference and sign an advanced directive. In contrast, patients who have no advance directive may either never think about their end-of-life care preference or may have thought about their end-of-life care preference and even talked about their decisions with their family members when their cognition function was not impaired. However, they did not complete the DNR document due to the context in Chinese culture, intrafamily or personal factors before they lose their mental capacity to make decisions. In Chinese culture, discussing death and end-of-life issues with patients with advanced illness is taboo [5], filial piety is valued, so children are not encouraged to disclose diseases to their parents or talk about hospice care [6]. This in turn means that most DNR decisions are taken by family members or surrogates of the patients at the point that patients are unable to provide consent by themselves, illustrated by a Taiwanese study showing that in patients who died of cancer, only 23% of DNR consent were provided by patients themselves, whereas the percentage of DNR orders signed by surrogates was 77% [7]. However, a DNR decision made by surrogates rather than patients themselves could indicate lower good death evaluation score, reflecting a poorer acceptance of death, limited arrangements of funeral care and poorer comfort care in the final three days of life [8]. Moreover, there is a gap in LST preferences between cancer patients and their family members, and family members are more likely to choose LST for the patients at the end-of-life [9]. The study revealed that, for example, only 33.3% of the terminal cancer patients opted for tube feeding, but up to 48.9% of the family members preferred tube feeding. This gap between patients and their family members may also exist in CKD population and influence the quality of patients’ end-of-life care.

For patients with advanced kidney disease, large cultural differences toward advanced directives were noted. In Taiwan, the uptake of advanced directives on end-of-life treatment preferences is low, with only 1.9% of patients with stage 5 CKD receiving renal replacement therapy signing one [10] compared to Canada where 38% of patients with stage 4 and 5 CKD had completed a personal directive [11]. Furthermore, the decision on whether or not to receive renal replacement therapy, and on the timing of withdrawing from dialysis is as crucial as the DNR decision in ACP for patients with advanced kidney disease [12]. Current evidence is insufficient to make conclusion on whether ACP has an impact on end-of-life treatments in hemodialysis patients [13]. Although it will be difficult to evaluate how the wider discussions of ACP influence the end-of-life care for these patients because the Patient Right of Autonomy Act was just implemented in 2019 [14], we have data from the year 2000 (the implementation of the Hospice and Palliative Care Act) to analyze DNR decision made by patients or surrogates is the alternative way to understand how patient autonomy play a role on end-of-life treatment.

Our study aims to evaluate the impact of patients’ autonomy on DNR and end-of-life treatments. Taiwan has the highest incidence and prevalence of treated end-stage renal disease (ESRD) in the world [2], so it is crucial to explore this issue in patients with advanced kidney disease. Key factors associated with different DNR consent types among patients with advanced kidney disease and association between DNR consent types and various life-prolonging treatments at the end-of-life will be investigated. We hypothesize that DNR consents signed by patients will use fewer life prolonging treatments compared to consents signed by surrogates.

## 2. Materials and Methods

### 2.1. Patient Selection

**Figure 1:**
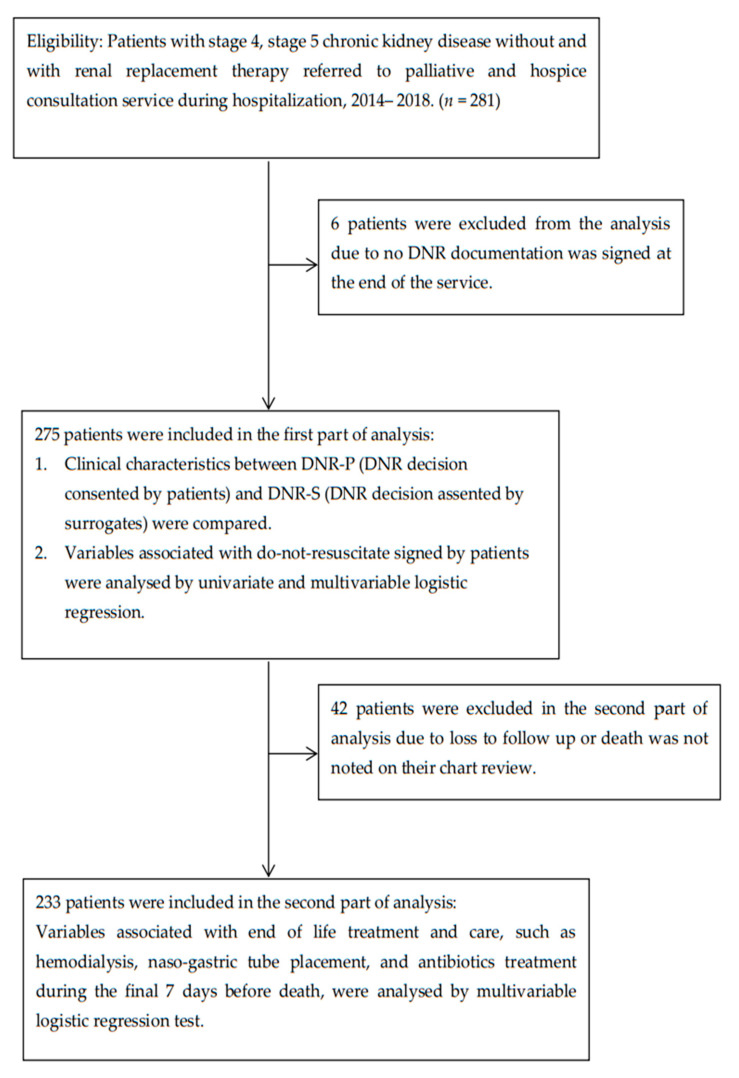
Patient enrollment and exclusion; DNR = do-not-resuscitate.

This retrospective study was conducted at a tertiary teaching hospital in Taiwan, and the diagram of two-stage patient selection is shown in Figure 1. Patients were selected using the following inclusion criteria: (i) receiving palliative and hospice care consultation service between January 2014 and December 2018, (ii) diagnosis of CKD stage 4 (estimated glomerular filtration rate (eGFR): 15–30 mL/min/1.73 m^2^), CKD stage 5 (eGFR less than 15 mL/min/1.73 m^2^ and not receiving renal replacement therapy) CKD, and stage 5 CKD with renal replacement therapy) before study enrollment, and (iii) age ≥20 years. We excluded those patients who did not have any DNR documentation signed by either by themselves or their surrogate decision-makers after palliative care consultation service. For the second part of the analysis, we excluded those who lost to follow-up or their death was not identified. This study protocol was approved by the Institutional Review Board of Kaohsiung Medical University Chung-Ho Memorial Hospital (KMUHIRB-E(II)-20200220).

### 2.2. Data Collection

Data were collected from medical record reviews, including age when receiving palliative care consultation service, sex, CKD stage, comorbidity, religious status, educational status, marital status, performance status at admission, residents in long-term care facilities, types of DNR consent and the date of fulfilling DNR documentation, date of palliative care consultation, date of death, and end-of-life treatment type in the final seven days before death (namely hemodialysis, nasogastric tube placement, and antibiotic use).

### 2.3. DNR Documentation

In our study, patients were classified into the following two groups according to their DNR documentation types: DNR-P (DNR document signed by patients) and DNR-S (DNR document signed by surrogates).

### 2.4. Statistical Analysis

We analyzed the prevalence of different DNR consent types (signed by patients or surrogates) and the covariates correlated with different DNR consent types among patients with advanced kidney disease. Cancer diagnosis was evaluated as an independent factor in terms of the strong association between cancer and palliative care [15]. We conducted additional analyses on whether these consents had an impact on various life-prolonging treatments at the end-of-life, such as tube feeding, antibiotic use, and continuation or termination of hemodialysis. Baseline and follow-up clinical characteristics were analyzed for correlation with DNR consent types using the chi-square or t test, and the 95% confidence interval (CI) was applied. We used logistic regression for univariate and mutivariable analyses to determine the most essential factors. The same method was used to analyze the influential factors of end-of-life treatments. MedCalc Statistical Software version 19.2.6 (MedCalc Software Ltd., Ostend, Belgium; viewed 16 March 2020, https://www.medcalc.org; 2020) was used for all statistical analyses.

## 3. Results

### 3.1. Patient Clinical Characteristics

A total of 281 terminally ill patients were eligible, of which 275 were included in the first part of the analysis. Six patients were excluded due to absence of DNR documentation. The mean age of our sample was 77.9 years. The mean age of the patients in the DNR-P and DNR-S groups was 80.2 and 77.3 years, respectively. Male gender proportion was 45% among all patients. It was 44% in the DNR-P group and 47% in the DNR-S group. There was no significant difference. As for CKD stage distribution, 40% of patients were at CKD stage 5 without RRT, followed by 33% for CKD stage 5 with RRT, and 28% for CKD stage 4. More than 40% of the study patients believed in Taiwan local religion, and more than 30% believed in Buddhism. Regarding religion, 15% of the study patients had no religion, and 6% believed in Christianism and Catholicism. More than half patients (55%) were married, and 40% lost their spouses. Very few patients were divorced (3%) or single (2%). As for education level, around half (51%) had medium education level, and 27% only had low education level. 22% of the patients had high education level. No significant differences were observed for CKD stages, religion, marital status and education level in the DNR-P and DNR-S groups (Table 1). The mean Charlson comorbidity index (CCI) was 8.8 and 8.0 in the DNR-P and DNR-S groups, respectively. The CCI score was significantly higher in the DNR-P group (*p*-value = 0.012). Moreover, the DNR-P group had proportionally more patients diagnosed with cancer concomitantly than in the DNR-S group (32% vs 17%, respectively). The difference was significant. Up to 74% of patients demonstrated poor performance status (3–4), with little difference between the two groups (79% vs 73%, respectively). In total, 33 patients resided in nursing homes, with most of these patients belonging to the DNR-S group. There was also significant different proportion of nursing home resident between the two groups (*p*-value = 0.027).

In the second part of the analysis, 233 patients were analysed for end-of-life treatments after 42 patients were excluded due to death not identified (Table 1, Follow-up characteristics). The demographic characteristics of these 233 patients were shown as Appendix A, Table A1. The mean survival after DNR consent singed and after palliative consultation was 134 days and 39 days respectively. The survival after DNR consent singed and after palliative consultation was significantly different between the DNR-P and DNR-S groups

In the final seven days before death, 32% of patients continued with hemodialysis. Fewer patients had hemodialysis in the final seven days in the DNR-P group compared to the DNR-S group (27% and 33%, respectively). For nasogastric (NG) tube placement, only 46% of patients had an NG tube placed in the DNR-P group compared to 83% of patients in the DNR-S group who underwent this procedure. Only half the DNR-P group (50%) were on antibiotics in the final seven days compared to 75% of the patients in the DNR-S group. The differences of NG tube placement and antibiotics use were significant between these two groups (*p*-value < 0.001, and 0.001 respectively).

### 3.2. Factors Related to DNR Decision

We used the logistic regression model to identify the influential factors related to DNR decision consented by patients. The results were demonstrated in Table 2. In the univariate analysis, there were three significant factors noted. Higher CCI (OR, 1.20; 95% CI, 1.04–1.39), and cancer diagnosis (OR, 2.19; 95% CI, 1.13–4.23) were positively related to have DNR signed by patients. In the opposite, nursing home residents (OR, 0.22; 95% CI, 0.05–0.95) were negatively related to DNR signed by themselves.

We use multivariable logistic regression to further analyse the factors. The OR of age for DNR consented by patients was 1.02 (95% CI, 0.98–1.06). As for the gender factor, the OR of being male was 0.75(95% CI, 0.37–1.52). When compared with CKD stage 4, the odds ratio of signing DNR consent by patients was 1.09 (95% CI, 0.51–2.33) in the CKD stage 5 without RRT groups, and was 0.70 (95% CI, 0.28–1.74) in the patients with CKD stage 5 with RRT. As for religion, the odds ratio of signing DNR consent by patients was 1.74 (95% CI, 0.62–4.83) in Taiwan local religion group, 1.37 (95% CI, 0.47–3.96) in Buddhism, and 1.93 (95% CI, 0.44–8.44) in the Christianism/Catholicism. When compared with married patients, the odds ratio of signing DNR consent by self was 0.80 (95% CI, 0.38–1.69) in the widowed group, 1.65 (95% CI, 0.29–9.29) in the divorced group, and 2.03 (95% CI, 0.17–23.61) in single patients. In the education level comparison, there was no significant difference between each level. We used the low education level group as reference, and the medium level group showed OR: 1.38 (95% CI, 0.61–3.11). The high education level group showed OR: 2.34 (95% CI, 0.84–6.54). The odds ratio for signing their own DNR consent in the patients with worse performance function was 1.37 (95% CI, 0.62–3.04).

Multivariable logistic regression revealed that nursing home residents were less likely to sign DNR consent by themselves (odds ratio [OR], 0.19; 95% CI, 0.04–0.88) (Table 2). The odds of having DNR consent signed by patients increased 15% with each point higher in Charlson comorbidity index (OR, 1.15; 95% CI, 0.98–1.37). The OR for the DNR consent signed by patients with a cancer diagnosis was 1.89 compared to patients without cancer (95% CI, 0.91–3.91). We noted a trend that increasing age was correlated with DNR decision consented by patients. Although the CI of the OR of this variable was across 1.0, the interval was narrow enough to indicate the tendency.

### 3.3. Factors Related to End-of-Life Treatments

Influential factors related to life-prolonging treatments during the final seven days before death is analyzed in Table 3, which demonstrates that the stage of chronic renal failure had a great impact on the decision to continue dialysis or not in the final seven days. Patients with stage 5 CKD with or without renal replacement therapy at baseline tended to have hemodialysis in the final seven days. The OR of having hemodialysis in the final seven days of life was 5.85 and 32.45 respectively in patients with stage 5 CKD without RRT and stage 5 CKD with RRT compared to patients with stage 4 CKD patients at baseline. Patients who believed in Taiwan local religion tended not to have hemodialysis before death (OR, 0.27; 95% CI, 0.09–0.78). Patients who believed in Buddhism also tended not to have hemodialysis before death, (OR, 0.33; 95% CI, 0.11–0.98). Patients who signed their own DNR consent had a lower risk of nasogastric NG tube placement (OR, 0.16; 95% CI, 0.07–0.34) and antibiotic treatment (OR, 0.33; 95% CI, 0.16–0.69) in the final seven days prior death than did DNR-S group patients. As for NG tube placement before death, patients with stage 5 CKD with RRT was less likely to use the tubes (OR, 0.37; 95% CI, 0.14–0.96) compared to patients with stage 4 CKD patients. Male patients had higher probability to have antibiotics use before death, the OR was 2.08 (95% CI, 1.02–4.24).

## 4. Discussion

This is the first investigation on how DNR consent signed by patients themselves could be vital in various end-of-life treatments in terminally ill patients with advanced kidney disease in the Asian context. All the enrolled patients received palliative care consultation service, which can increase awareness of diagnosis and prognosis among patients and their families before death [16]. However, the end-of-life treatments varied greatly between the study patients in DNR-P and DNR-S groups. The DNR consent signed by patients, documenting their preferences for withholding cardiopulmonary resuscitation (CPR), is a core element of advanced directives and a strong measure of patient autonomy on their end-of-life preferences. Palliative care consultation service may offer the opportunity to enhance the conversation among patients who signed their DNR consent, their family members, and clinicians on preferences of end-of-life treatments in addition to CPR choices. This potentially leads to less utilization of other life-prolonging treatments at the end of life in the DNR-P group.

Decision-making and autonomy for end-of-life care vary considerably between different cultures and societies [17,18]. In the Taiwanese cultural context, filial piety is valued highly, and most physicians’ clinical dilemmas involve family-centered decision-making situations [6]. Family meetings are an essential element of palliative care consultation service and may offer valuable opportunities to communicate for a consensus. The end-of-life treatment and care plans of those who signed their DNR consent themselves could be further discussed and understood by patients’ families or surrogates through family meetings. This process may assist physicians and increase the family’s respect for patient autonomy, thus paving the way to design treatment plans following patient preferences. This process also reflects the value of relational autonomy which is adopted in Asian countries [18].

Information on how advanced care planning or DNR consent influences end-of-life treatments in patients with advanced kidney disease is scarce. Kirchhoff et al. revealed that the patients with ESRD were significantly more likely to withdraw from dialysis if intervened with patient-centered advance care planning (PC-ACP; 38% with PC-ACP vs. 17% with usual care) [19]. The authors also indicated that patients under PC-ACP intervention tended to stop all treatments if the survival chances were low (73% with PC-ACP vs. 55% with usual care). However, the authors did not include other end-of-life treatment issues, such as those related to antibiotics use or nasogastric tube feeding. Our research provides evidence for patients with advanced CKD that although they all received palliative care, DNR consent signed by patients showed a lower risk of life-sustaining treatments at the end-of-life than DNR signed by surrogates. In the Chinese culture, discussion on DNR and disclosure of terminal disease is taboo because it is associated with bad luck or a short survival period [5]. Our findings revealed that the DNR-P group had a significantly longer survival period after palliative care consultation than the DNR-S group even though a higher proportion of patients from the DNR-S were using antibiotics and nasogastric tube feeding to prolong life in the final seven days of life.

As for hemodialysis in the final seven days of life, the status of dialysis at admission played a more important role, whereas the types of DNR consent did not make difference. Withdrawing hemodialysis is more difficult in decision-making by patients, family members, and medical professionals than withholding hemodialysis [6]. Withdrawing long-term hemodialysis at the end-of-life is believed to have more impact on actively causing patients’ dying than withdrawing or withholding antibiotics treatment or tube-feeding. Therefore, even patients in DNR-P group may have dilemma in whether to stop dialysis in the final seven days of life. Financial burden was reported as a factor of withholding or withdrawing life-sustaining treatments in some countries [20]. The situation in Taiwan is different because hemodialysis is fully reimbursed by National Health Insurance without out-of-pocket payment from patients, making the influence of financial issues much less substantial in the decision-making about hemodialysis.

The influence of age on implementations of DNR consent or end-of-life planning is controversial in different population and culture. A study in Taiwan revealed that younger patients with terminally-ill cancer tended to have DNR consent signed by themselves instead of surrogates [8]. However, a US study indicated that advanced age was significantly associated with DNR orders in patients with heart failure [21]. In the current study, we noted that increasing age was slightly related to DNR decision consented by patients themselves in the advanced kidney disease population, in contrast to the results observed in patients with cancer in Taiwan.

Patients with a high Charlson comorbidity index and cancer diagnosis tended to sign DNR on their own in our study. A previous meta-analysis focusing on elderly patients (age ≥ 80 years) revealed that DNR orders were positively related to multimorbidity [22]. Another study on inpatients with heart failure indicated that a high Charlson comorbidity index was related to an increase in DNR orders [21]. In addition, a study on patients with dementia indicated that a cancer diagnosis demonstrated protective effects against the use of life-prolonging treatments [15]. These two factors (high Charlson comorbidity index and cancer diagnosis), however, did not reach significance in the multivariable logistic analysis. Therefore, further investigation on whether multiple comorbidities and cancer diagnosis facilitate patients with advanced kidney disease to prepare for end-of-life issues in advance is warranted.

Nursing home residents may display a low likelihood of signing DNR consent by themselves. One US study documenting multiple nursing homes in California demonstrated that only 28.6% of Physician Orders for Life Sustaining Treatment were signed by the residents; 62.5% were signed by only the resident’s proxy, with 8.9% not signed [23]. An investigation on nursing home residents in Taiwan revealed that only 16.4% had DNR directives and 91% of the DNR directives were signed by surrogates [24]. Our findings were consistent with those of earlier studies, which may be partially explained by the family-oriented culture of Asian countries [25]. Furthermore, elderly patients in nursing homes may have less self-autonomy, complicated by the concept of filial piety in Chinese culture, where family surrogates make more of the medical decisions for the residents [18]. No differences were observed between nursing home residents and patients cared at home in our study regarding end-of-life treatments including hemodialysis, nasogastric tube feeding, and antibiotic use in the final seven days.

### Strengths and Limitations

This study provides crucial evidence on the end-of-life issues among patients with advanced kidney disease because the participants were recruited from Taiwan which has a higher incidence and prevalence of treated end-stage renal disease (ESRD) in the world. In addition, National Health Insurance in Taiwan is a nationwide single-payer healthcare scheme which covers all kind of renal replacement therapies, life-sustaining treatments, and palliative care for people with advanced kidney disease. The influence of the economic burden for patients and family members on medical decision making is much less.

Our study has some limitations. First, this was a retrospective observational study, so we can only establish a correlation but not a causal relationship between DNR consent type and end-of-life treatments. Second, the process of DNR signing and real conversations on end-of-life care preference were not recorded, and there was insufficient information in the chart review about the cognitive function of the patients when the DNR document was signed. Third, the treatments in the final seven days may be required because of complicated clinical conditions, not just patients’ preferences. Furthermore, the association identified in our study may be only relevant in the Chinese culture or some other East Asian countries, and the generalizability in the Western culture needs further validation. Additional prospective studies examining the concordance between patient preferences and the final end-of-life treatment are needed, in which future work should look at evaluating the different effects of life-prolonging treatments and end-of-life care between advanced care planning and DNR consent discussion in palliative care consultation among patients with advanced kidney disease in the Asian cultural context within the specific legal framework in Taiwan.

In conclusion, the DNR consent signed by patients had a great impact on end-of-life treatments among terminally ill patients. Compared to patients with advanced kidney disease whose DNR decision was assented by surrogates, those who signed their own DNR consent may demonstrate better autonomy in medical decision making during the palliative care consultation and result in a lower risk of nasogastric tube use and antibiotics treatment in the final seven days before death. This suggests that DNR consent signed by patients and enhanced by palliative care consultation may be crucial in providing better outcomes at the end-of-life in clinical practice for patients with advanced kidney disease.

## Figures and Tables

**Table 1:** Baseline and follow-up clinical characteristics in patients with advanced kidney disease receiving palliative care consultation during January 2014–December 2018.

Baseline Characteristics	All Patients	DNR-P	DNR-S	*p*-Value
	*n* = 275	*n* = 57	*n* = 218	
Age in years, mean (SD)	77.9 (±11.0)	80.2 (±8.9)	77.3 (±11.4)	0.076
Gender (Male)	127 (45%)	25 (44%)	102 (47%)	0.693
CKD stage				
4	76 (28%)	17 (30%)	59 (27%)	0.509
5 without RRT	109 (40%)	25 (44%)	84 (39%)	
5 with RRT	90 (33%)	15 (26%)	75 (34%)	
Charlson Comorbidity Index	8.2	8.8	8	0.012
	(7.9–8.4)	(8.2–9.3)	(7.7–8.3)	
With Cancer diagnosis	56 (20%)	18 (32%)	38 (17%)	0.018
Religion				
Taiwan local religion	119 (43%)	27 (47%)	92 (42%)	
Buddhism	96 (35%)	19 (33%)	77 (35%)	0.538
Christianism/Catholicism	16 (6%)	5 (9%)	11 (5%)	
None	41 (15%)	6 (11%)	35 (16%)	
Others	3 (1%)	0 (0%)	3 (1%)	
Marital status				0.994
Married	151 (55%)	31 (54%)	120 (55%)	
Widowed	109 (40%)	23 (40%)	86 (39%)	
Divorced	9 (3%)	2 (4%)	7 (3%)	
Single	6 (2%)	1 (2%)	5 (2%)	
Education				0.551
Low	75 (27%)	13 (23%)	62 (28%)	
Medium	140 (51%)	29 (51%)	111 (51%)	
High	60 (22%)	15 (26%)	45 (21%)	
Performance status ^1^				0.323
1–2	72 (26%)	12 (21%)	60 (28%)	
3–4	203 (74%)	45 (79%)	158 (73%)	
Nursing home residents	33 (12%)	2 (4%)	31 (14%)	0.027
**Follow-up Characteristic ^2^**	**All Patients**	**DNR-P**	**DNR-S**	***p*-Value**
	***n* = 233**	***n* = 48**	***n* = 185**	
Survival after DNR consent singed (days)	134	271	98	<0.001
	(±300)	(±419)	(±250)	
Survival after palliative consultation (days)	39	80	28	<0.001
	(±90)	(±139)	(±68)	
Treatments in the last seven days:				
Hemodialysis	74 (32%)	13 (27%)	61 (33%)	0.436
Nasogastric tube placement	175 (75%)	22 (46%)	153 (83%)	<0.001
Antibiotics use	162 (70%)	24 (50%)	138 (75%)	0.001

Results are presented as mean with standard deviation, or number (percentage). CKD, chronic kidney disease; RRT, renal replacement therapy; DNR-P: do-not-resuscitate document signed by patients; DNR-S: do-not-resuscitate document signed by surrogates. ^1^ Performance status score 1–4 represents the following: 1—Symptomatic but completely ambulatory; 2—Symptomatic, <50% in bed during the day; 3—Symptomatic, >50% in bed, but not bedbound; 4—Bedbound. ^2^ All the patients were followed up until death; 42 patients were excluded due to loss follow-up or death was not identified upon chart.

**Table 2:** Factors related to do-not-resuscitate consent signed by patients themselves in patients with advanced kidney disease.

Variable	Univariate Analysis	*p*-Value	Multivariable Analysis	*p*-Value
	OR (95% CI)		OR (95% CI)	
Age	1.03 (0.99–1.06)	0.078	1.02 (0.98–1.06)	0.285
Gender				
Female	Reference		Reference	
Male	0.89 (0.49–1.60)	0.693	0.75 (0.37–1.52)	0.425
CKD stage				
4	Reference		Reference	
5 without RRT	1.03 (0.51–2.08)	0.928	1.09 (0.51–2.33)	0.82
5 with RRT	0.69 (0.32–1.50)	0.355	0.70 (0.28–1.74)	0.442
Charlson Comorbidity Index	1.20 (1.04–1.39)	0.013	1.15 (0.98–1.37)	
				0.092
With cancer diagnosis				
No	Reference		Reference	
Yes	2.19 (1.13–4.23)	0.002	1.89 (0.91–3.91)	0.088
Religion				
None	Reference		Reference	
Taiwan local religion	1.71 (0.65–4.50)	0.276	1.74 (0.62–4.83)	0.290
Buddhism	1.44 (0.53–3.92)	0.476	1.37 (0.47–3.96)	0.566
Christianism/Catholicism	2.65 (0.68–10.40)	0.162	1.93 (0.44–8.44)	0.385
others	0.0000	0.998	0.0000	0.998
Marital status				
Married	Reference		Reference	
Widowed	1.04 (0.56–1.90)	0.911	0.80 (0.38–1.69)	0.563
Divorced	1.11 (0.22–5.59)	0.903	1.65 (0.29–9.29)	0.573
Single	0.77 (0.09–6.87)	0.818	2.03 (0.17–23.61)	0.573
Education				
Low	Reference		Reference	
Medium	1.25 (0.60–2.57)	0.552	1.38 (0.61–3.11)	0.436
High	1.59 (0.69–3.67)	0.277	2.34 (0.84–6.54)	0.105
Performance status ^1^				
1–2	Reference		Reference	
3–4	1.42 (0.71–2.88)	0.324	1.37 (0.62–3.04)	0.440
Nursing home residents				
No	Reference		Reference	
Yes	0.22 (0.05–0.95)	0.042	0.19 (0.04–0.88)	0.033

CI, confidence interval; OR, odds ratio; CKD, chronic kidney disease; RRT, renal replacement therapy ^1^ Performance status score 1–4 represents the following: 1—Symptomatic but completely ambulatory; 2—Symptomatic, <50% in bed during the day; 3—Symptomatic, >50% in bed, but not bedbound; 4—Bedbound.

**Table 3:** Factors related to end-of-life treatments in the final seven days before death in patients with advanced kidney disease.

Variable	Hemodialysis		Nasogastric Tube Placement		Antibiotics Use	
	OR (95% CI)	*p*-Value	OR (95% CI)	*p*-Value	OR (95% CI)	*p*-Value
Age	0.97 (0.93–1.01)	0.096	0.96 (0.92–1.001)	0.06	1.01 (0.97–1.04)	0.770
Gender						
female	Reference		Reference		Reference	
Male	1.20 (0.55–2.62)	0.654	0.69 (0.32–1.48)	0.34	2.08 (1.02–4.24)	0.045
CKD stage						
4	Reference		Reference		Reference	
5 without RRT	5.85 (1.77–19.33)	0.004	0.70 (0.29–1.68)	0.425	0.94 (0.44–2.03)	0.881
5 with RRT	32.45 (9.28–113.49)	<0.001	0.37 (0.14–0.96)	0.042	1.20 (0.51–2.82)	0.673
Charlson Comorbidity Index	1.06 (0.87–1.29)	0.583	0.91 (0.75–1.10)	0.334	0.85 (0.72–1.02)	0.074
With cancer diagnosis						
No	Reference		Reference		Reference	
Yes	1.20 (0.50–2.88)	0.677	1.11 (0.45–2.71)	0.827	1.79 (0.76–4.21)	0.184
Religion						
None						
Taiwan local religion	Reference		Reference	0.604	Reference	
Buddhism	0.27 (0.09–0.78)	0.016	1.45 (0.51–4.08)	0.487	1.62 (0.61–4.28)	0.334
Christianism/	0.33 (0.11–0.98)	0.047	1.33 (0.46–3.85)	0.604	1.10 (0.41–2.91)	0.849
Catholicism	1.76 (0.31–9.98)	0.525	2.14 (0.38–11.93)	0.385	0.55 (0.12–2.59)	0.452
Marital status						
Married	Reference				Reference	
Widowed	0.55 (0.24–1.24)	0.149	Reference	0.063	0.51 (0.25–1.04)	0.074
Divorced	1.40 (0.23–8.56)	0.719	0.47 (0.21–1.04)	0.609	0.40 (0.0–2.27)	0.301
Single	2.35 (0.11–51.88)	0.587	1.85 (0.18–19.33)	0.999	0.61 (0.05–7.54)	0.700
Education						
Low	Reference		Reference		Reference	
Medium	0.86 (0.36–2.08)	0.744	0.43 (0.18–1.03)	0.059	0.50 (0.23–1.08)	0.077
High	0.58 (0.19–1.75)	0.338	0.57 (0.19–1.74)	0.322	0.97 (0.3–1.66)	0.952
Performance status ^1^						
1–2	Reference		Reference		Reference	
3–4	0.59 (0.24–1.46)	0.257	1.01 (0.43–2.36)	0.98	0.76 (0.34–1.66)	0.487
Nursing home residents						
No	Reference		Reference		Reference	
Yes	0.66 (0.22–1.93)	0.446	2.78 (0.72–10.74)	0.138	1.97 (0.67–5.75)	0.215
DNR consent						
Signed by surrogates	Reference		Reference		Reference	
Signed by patients	0.92 (0.38–2.23)	0.854	0.16 (0.07–0.34)	<0.001	0.33 (0.16–0.69)	0.003

CI, confidence interval; OR, odds ratio; ESRD, end-stage renal disease; CKD, chronic kidney disease; DNR, do-not-resuscitate. ^1^ Performance status score 1–4 represents the following: 1—Symptomatic but completely ambulatory; 2—Symptomatic, <50% in bed during the day; 3—Symptomatic, >50% in bed, but not bedbound; 4—Bedbound.

## Data Availability

The data for this study was obtained from the electronic chart systems in Kaohsiung Medical University Hospital. The permission of data availability was limited to current study only according to the ethical approval. Data are however can be checked for any researcher who may concern about its reliability upon reasonable request to the Department of Family Medicine or Institutional Review Board of Kaohsiung Medical University Chung-Ho MemorialHospital, Taiwan.

## Appendix A

**Table A1:** Follow-up clinical characteristics in patients with advanced kidney disease receiving palliative care consultation during January 2014–December 2018.

Follow-Up Characteristic	All Patients	DNR-P	DNR-S	*p*-Value
	*n* = 233	*n* = 48	*n* = 185	
Age in years, mean (SD)	77.6(±10.8)	79.6(±9.3)	77.0(±11.2)	0.152
Gender (Male)	105(45%)	20(42%)	85(46%)	0.596
CKD stage				
4	66(28%)	15(31%)	51(28%)	0.613
5 without RRT	90(39%)	20(42%)	70(38%)	
5 with RRT	77(33%)	13(27%)	64(35%)	
Charlson Comorbidity Index	8.2	8.8	8.0	0.023
	(7.9–8.4)	(8.2–9.4)	(7.7–8.3)	
With Cancer diagnosis	44(19%)	14(29%)	30(16%)	0.042
Religion				
Taiwan local religion	100(43%)	24(50%)	76(41%)	
Buddhism	84(36%)	16(33%)	68(37%)	0.446
Christianism/Catholicism	13(6%)	4(8%)	9(5%)	
None	35(15%)	4(8%)	31(17%)	
Others	1(<1%)	0(0%)	1(<1%)	
Marital status				0.949
Married	131(56%)	27(56%)	104(56%)	
Widowed	91(39%)	18(38%)	73(40%)	
Divorced	7(3%)	2(4%)	5(3%)	
Single	4(2%)	1(2%)	3(2%)	
Education				0.830
Low	63 (27%)	12(25%)	51(28%)	
Medium	119 (51%)	24(50%)	95(51%)	
High	51 (22%)	12(25%)	39(21%)	
Performance status ^1^				0.317
1–2	67(29%)	11(23%)	56(30%)	
3–4	166(71%)	37(77%)	129(70%)	
Nursing home residents	28(12%)	2(4%)	26(14%)	0.061

Results are presented as mean with standard deviation, or number (percentage). CKD, chronic kidney disease; RRT, renal replacement therapy; DNR-P: do-not-resuscitate document signed by patients; DNR-S: do-not-resuscitate document signed by surrogates. ^1^ Performance status score 1–4 represents the following: 1—Symptomatic but completely ambulatory; 2—Symptomatic, <50% in bed during the day; 3—Symptomatic, >50% in bed, but not bedbound; 4—Bedbound.
